# Rural-urban differences in the association between individual, facility, and clinical characteristics and travel time for cancer treatment

**DOI:** 10.1186/s12889-020-8282-z

**Published:** 2020-02-06

**Authors:** Joel E. Segel, Eugene J. Lengerich

**Affiliations:** 1 Department of Health Policy and Administration, Pennsylvania State University, 504 S Ford Building, University Park, PA 16802 USA; 2 Penn State Cancer Institute, Hershey, PA USA; 3 Department of Public Health Sciences, Pennsylvania State University College of Medicine, Hershey, PA USA

**Keywords:** Rural-urban disparities, Health services accessibility, Rural health services, Cancer, Travel time

## Abstract

**Background:**

Greater travel time to cancer care has been identified as a potential barrier to care as well as associated with worse health outcomes. While rural cancer patients have been shown to travel farther for care, it is not known what patient, facility, and clinical characteristics may differentially be associated with greater roundtrip travel times for cancer patients by rurality of residence. Identifying these factors will help providers understand which patients may be most in need of resources to assist with travel.

**Methods:**

Using 2010–2014 Pennsylvania Cancer Registry data, we examined the association between patient, facility, and clinical characteristics with roundtrip patient travel time using multivariate linear regression models. We then estimated separate models by rural residence based on the Rural-Urban Continuum Code (RUCC) of a patient’s county of residence at diagnosis to understand how the association of each factor with travel time may vary for patients separated into metro residents (RUCC 1–3); and two categories of non-metro residents (RUCC 4–6) and (RUCC 7–9).

**Results:**

In our sample (*n* = 197,498), we document large differences in mean roundtrip travel time—mean 41.5 min for RUCC 1–3 patients vs. 128.9 min for RUCC 7–9 patients. We show cervical/uterine and ovarian cancer patients travel significantly farther; as do patients traveling to higher volume and higher-ranked hospitals.

**Conclusions:**

To better understand patient travel burden, providers need to understand that factors predicting longer travel time may vary by rurality of patient residence and cancer type.

## Background

Increasingly cancer studies have identified distance to care as an important measure of access to care and as a result impacts health outcomes [1–4]. Previous studies have examined the association of travel distance [1, 3] or time [2, 4] with various cancer-related factors including stage at diagnosis, [5–8] type of treatment, [8–13] and treatment outcome [9, 14–17]. One set of studies has examined the association between provider availability and patient travel times including one study that found less than half of the population of the US lives within 1 hour of a National Cancer Institute (NCI)-designated cancer center and over 90% live within 1 hour of specialty oncology care [3]. Another study found that chemotherapy patients living in areas with no oncologist traveled significantly farther [1].

A second set of studies have examined the relationship between distance to facilities and treatment type. Several studies found that living farther from radiation treatment facilities decreased the likelihood of receiving radiation therapy for breast [9, 10] and prostate cancer [12]. Similarly, two studies found that greater distance to the nearest chemotherapy provider decreased the likelihood of colon cancer patients receiving adjuvant chemotherapy [11, 13]. These studies illustrate the concern that greater distance may alter treatment choice and as a result may have a negative effect on outcomes.

A third set of studies have examined whether travel time affects outcomes. One study reported no difference in quality of care (time to cystectomy or use of neoadjuvant chemotherapy) for bladder cancer patients but did report that greater travel distance was associated with significantly increased 90-day mortality [16]. Conversely, several studies have reported greater distance to be associated with improved mortality [14, 18]. However, an analytic difficulty and possible explanation for these results is that patients able to travel longer distances may be healthier than those unable to travel longer distances.

Finally, several studies have examined how travel time may affect cancer patients’ choice of hospital. Several studies have focused on rural Medicare cancer patients, including one that found 60% of rural Medicare patients went to the nearest hospital regardless of size [19] and others that found rural Medicare patients were more likely to choose teaching hospitals and hospitals with a wider range of services [19, 20]. Additionally, a study of gastric cancer patients undergoing gastrectomy found that while rural patients were significantly more likely to go to the nearest hospital, they preferred teaching hospitals and higher volume hospitals [21]. A study of Iowa patients undergoing radiation therapy found greater travel times for younger, male, and rural patients [22]. Underscoring the complexity of the relationship between rurality, travel time, and receipt of care prior work has shown that patients living in rural areas may see fewer specialists and more generalists, [23] but that travel time can also be just one factor in explaining rural-urban disparities in care [24].

While these studies highlight factors related to travel and hospital choice, they were largely restricted to patients exclusively from rural areas or patients undergoing a specific treatment. Therefore, they were not able to examine factors that might differentially affect patients living in rural areas compared to urban areas or that may affect the broader spectrum of cancer patients. Answers to these questions will help cancer centers develop effective travel-sensitive clinical outreach throughout their catchment area and may help providers better understand differences in patient needs. While, admittedly, some programs currently exist, [25–27] relatively few have been systematically described and evaluated in the literature.

Thus, the objectives of this study were to: 1) estimate travel times to hospitals using a population-based approach for all types of cancer patients and to examine factors associated with greater travel times, and [2] estimate whether these associations were different between patients residing in rural areas compared to metro areas. Pennsylvania, the location for this study, is the 5th most populous U.S. state with about 12% of its nearly 80,000 annual cancer patients residing in rural areas [28]. Furthermore, four NCI-designated cancer centers exist in Pennsylvania; although they are exclusively located in metro areas in southeastern and southwestern Pennsylvania.

## Methods

### Study data

The study used a population-based retrospective design beginning with all invasive cancer cases initially diagnosed between 2010 and 2014 within the Pennsylvania Cancer Registry database, with the exception of less than 3% of cases omitted due to interstate data exchange, Health Insurance Portability and Accountability Act (HIPAA), or being Veterans Affairs records. We restricted analyses to Pennsylvania residents age 18 or older with a diagnosis of invasive cancer, receiving treatment in Pennsylvania, and who had non-missing data on residential longitude and latitude (< 0.1% were missing residential location). In addition, to ensure that we restricted analyses to those receiving treatment at the observed facility, we limited our sample to analytic cases and excluded cases identified on death certificate or autopsy only, and those who received only diagnosis and no treatment at the specific facility. To further focus on treatment, in sensitivity analyses, we restricted to facilities that had at least 500 cancer cases over the 5-year period.

Data included patient demographics, health insurance, patient residence location at diagnosis, primary cancer site, stage of diagnosis, the name of each facility where the patient was treated, as well as treatment modality and date of treatment. For each case, we estimated patient travel time using the Stata command osrmtime, [29] which uses the Open Source Routing Machine and OpenStreetMap data to calculate the shortest travel time between two geographic points. We input patients’ latitude and longitude, calculated by the Cancer Registry based on the patient’s address at diagnosis including PO boxes or rural routes; together with facility name and the associated facility-level longitude and latitude data from Medicare [30] to estimate travel time. While the program does not account for variations in travel time that could arise due to weather or changing traffic patterns, it is an estimate of travel time rather than distance “as the crow flies”. From this measure of travel time, we created the primary outcome of interest: roundtrip travel time measured in minutes.

### Statistical analysis

We estimated a series of multivariate linear regression models with roundtrip travel time as the outcome. For each linear model, we also examined how individual-, clinical-, and hospital-level factors were associated with travel time. Specifically, we examined the association between roundtrip travel time and individual factors including: age (ages 40–64, ages 65 or older with under age 40 as the reference group), race (non-white and missing race with white as the reference group), Hispanic ethnicity, insurance type (uninsured, Medicaid, Medicare, dual Medicare-Medicaid, and other insurance with private insurance as the reference group), and rural/urban county-level residence using the United States Department of Agriculture Economic Research Service Rural-Urban Continuum Codes (RUCC) [31]. Based on the prior literature [32–34], we defined three categories of rural/urban residence based on the RUCC of residence at diagnosis. The categories included metro residence (RUCC 1–3) as well as two categories of non-metro or rural residence: RUCC 4–6 and RUCC 7–9. We particularly focused on RUCC of 4 or greater given the emphasis on these rural areas by the NCI [35]. Finally, we note that in Pennsylvania no county has a RUCC of 5.

We also examined clinical factors including primary cancer site defined using ICD-O-3 codes from the NCI [36] (lung/bronchus, colorectal, prostate, female breast, cervical/uterine, oral/pharyngeal, ovarian, thyroid, melanoma, other urological, and other digestive, with other types as the reference group—specific codes available upon request) and whether the cancer was diagnosed at the regional stage, distant stage, or of unknown locality with local stage as the reference group. Finally, we included the following hospital-level measures: 2016 US News and World Report (USNWR) oncology care score or whether the score was missing (i.e., typically lower volume facilities) [37], the distance in minutes to the nearest facility, and total cancer care volume from 2010 to 2014. While the USNWR scores are not a validated quality measure, we included them because they are a widely available, public ranking that all patients can access. The scores have a single ranking value making them easily understood and from a widely respected source that provides commonly used rankings across a number of sectors including health care and education [38]. So while the measure may imperfectly measure quality, it is a measure that would be widely available for patients. We estimated these models by each of the three categories including: metro, rural RUCC 4–6, and rural RUCC 7–9 to analyze whether the factors affecting travel time differed between non-metro and metro residents. To compare estimates across the stratified models, we used estimates from seemingly unrelated regression models to compare coefficients across models and to test for statistically significant differences.

To control for the possibility of visits to multiple facilities for the same tumor, we assigned each facility an indicator based upon the chronological order of the visit (i.e., a 1 for the first facility visited, a 2 for the second, etc.) using a two-step process. We first assigned the facility that diagnosed the patient as the initial facility. For patients who visited three or more facilities we then used the earliest listed visit date to order the subsequent facilities. We then controlled for this chronological facility number in all analyses. In addition, to account for the fact that individual patients may be observed more than once in the data set, we clustered all standard errors at the individual patient level. Finally, in a sensitivity analysis, we restricted the sample to only the earliest facility listed for each individual patient.

In addition to the sensitivity analysis restricting analyses to just the earliest facility for each individual, we ran three additional sets of sensitivity analyses. First, we included all facilities seeing at least 500 patients over the 5-year study window. Second, we re-estimated all models without controlling for travel time to the nearest hospital. Finally, we estimated models that controlled for whether the treatment facility was a NCI-designated facility. Specifically, these were all cases for which the patient was seen at the University of Pennsylvania hospitals, Fox Chase Cancer Center, Thomas Jefferson University, or the University of Pittsburgh Medical Center’s Magee Women’s or Shadyside locations. All analyses were estimated using Stata version 14.2.

## Results

We identified 197,498 cases, including 175,184 cases with a metro residence (88.7%), 19,346 with a rural RUCC 4–6 residence (9.8%), and 2968 with a rural RUCC 7–9 residence (1.5%). Mean age was similar, although slightly lower among metro patients ranging from 66.0 for metro residents to 67.0 for rural RUCC 7–9 residents (Table 1). For the other patient, facility, and clinical characteristics, we found significant mean differences between residents of metro and non-metro areas. Non-metro residents (including both rural RUCC 4–6 and rural RUCC 7–9) were more likely to be male, white, and Medicare or dual-eligible insurance holders. In addition, they were more likely to live farther from the nearest facility and go to facilities with lower USNWR scores or facilities that did not have scores.

**Table 1:** Summary statistics overall and by rurality of residence location at diagnosis

	Metro	Rural RUCC 4–6	*P value* (comparison to metro)	Rural RUCC 7–9	*P value* (comparison to metro)	*P value* (compare rural RUCC 4-6to rural RUCC 7–9)
	*N* = 175,184	*N* = 19,346		*N* = 2968		
Mean age (years)	66.0	66.5	< 0.001	67.0	< 0.001	0.05
Female	51.2%	48.2%	< 0.001	47.4%	51.2%	0.43
White	86.7%	98.3%	< 0.001	98.0%	86.7%	0.24
Non-white	12.4%	1.4%	< 0.001	1.3%	12.4%	0.74
Hispanic	1.72%	0.35%	< 0.001	0.40%	< 0.001	0.62
Uninsured	1.0%	1.1%	0.40	1.0%	0.96	0.71
Private insurance	36.8%	32.0%	< 0.001	29.3%	< 0.001	0.00
Medicaid	5.7%	5.6%	0.43	5.2%	0.21	0.37
Medicare	44.7%	49.5%	< 0.001	54.4%	< 0.001	< 0.001
Dual-eligible	2.9%	4.2%	< 0.001	5.6%	< 0.001	< 0.001
Other insurance	8.9%	7.6%	< 0.001	4.5%	< 0.001	< 0.001
Travel time to nearest facility (minutes)	19.3	31.8	< 0.001	42.7	< 0.001	< 0.001
Mean number of facilities visited	1.0	1.0	0.03	1.0	0.02	0.11
US News hospital score	26.9	17.3	< 0.001	15.3	< 0.001	< 0.001
US News hospital score missing	26.9%	45.5%	< 0.001	56.6%	< 0.001	< 0.001
Volume of cases 2010–2014 at facility attended (in 100’s)	49.5	44.6	< 0.001	43.3	< 0.001	0.20
Lung/bronchus	17.9%	17.6%	0.47	16.5%	0.06	0.14
Colorectal	12.5%	13.8%	< 0.001	14.1%	0.01	0.67
Prostate	13.8%	13.8%	0.96	16.9%	< 0.001	< 0.001
Female breast	17.8%	15.8%	< 0.001	16.6%	0.08	0.25
Cervical/uterine	0.9%	0.8%	0.56	0.5%	0.02	0.04
Oral/pharyngeal	3.0%	3.2%	0.13	2.8%	0.68	0.34
Ovarian	1.9%	1.8%	0.59	1.7%	0.56	0.73
Thyroid	5.5%	5.3%	0.31	5.5%	0.98	0.68
Melanoma	5.7%	6.0%	0.07	7.0%	0.00	0.03
Other urological	9.5%	10.5%	< 0.001	9.5%	0.93	0.08
Other digestive	11.6%	11.3%	0.28	8.8%	< 0.001	< 0.001
Local	52.1%	52.1%	0.90	55.1%	0.00	0.00
Regional	24.1%	23.9%	0.66	21.2%	< 0.001	0.00
Distant	21.5%	21.5%	0.96	20.2%	0.09	0.11
Unknown stage	2.2%	2.5%	0.06	3.5%	< 0.001	< 0.001
Chemotherapy	26.0%	24.8%	< 0.001	21.0%	< 0.001	< 0.001
Radiation therapy	25.4%	23.5%	< 0.001	23.9%	0.06	0.64
Surgery	66.5%	67.7%	< 0.001	69.6%	< 0.001	0.04
Immunotherapy	0.9%	0.7%	< 0.001	1.0%	0.69	0.03
Hormone therapy	15.7%	14.5%	< 0.001	16.8%	0.10	< 0.001

^a^For test of differences between rural-urban category of residence, *P value* is for t-test for continuous variables and Chi-squared test for categorical variables

We found substantial variation in mean roundtrip travel times across a number of characteristics both within rural-urban categories and across categories as shown in Table 2. We found mean roundtrip travel times of 41.5 min for metro residents, rising to 95.6 min for rural RUCC 4–6 residents (*p* < 0.001 compared to metro residents) and to 128.9 min for rural RUCC 7–9 residents (*p* < 0.001 compared to metro residents). In addition, across each group we found that non-elderly patients, males, white patients, those with private insurance, those living further from the nearest facility, those attending facilities with higher USNWR scores, those attending higher volume facilities, and patients receiving surgical care all had longer unadjusted travel times to care. Clinically, results were mixed. Patients with local stage cancers appeared to travel slightly longer for those living in metro areas, whereas those with regional cancer traveled farther among patients living in non-metro areas. We observed consistently shorter travel times for patients with breast or colorectal cancer across each rural-urban category. However, while prostate, melanoma, and oral/pharyngeal cancer patients appeared to travel farther in metro areas, patients with cervical/uterine, ovarian, and thyroid cancers appeared to travel farthest among patients living in non-metro areas.

**Table 2:** Unadjusted mean round trip travel times (in minutes) by rural-urban category and individual, provider, and clinical characteristic

	All (*n* = 197,498)	Metro (*n* = 175,184)	Rural RUCC 4–6 (*n* = 19,346)	*P value* (metro vs. rural RUCC 4–6)	Rural RUCC 7–9 (*n* = 2968)	*P value* (metro vs. rural RUCC7–9)	*P value* (rural RUCC 4–6 vs. rural RUCC 7–9)
Overall	48.1	41.5	95.6	< 0.001	128.9	< 0.001	< 0.001
Ages < 40	56.4	48.7	117.6	< 0.001	150.7	< 0.001	0.001
Ages 40–64	52.6	45.4	107.5	< 0.001	150.9	< 0.001	< 0.001
Ages 65+	44.3	38.1	86.5	< 0.001	114.4	< 0.001	< 0.001
Male	51.8	44.7	99.3	< 0.001	139.0	< 0.001	< 0.001
Female	44.5	38.4	91.6	< 0.001	117.6	< 0.001	< 0.001
White	51.1	44.0	95.8	< 0.001	129.0	< 0.001	< 0.001
Non-white	24.4	23.6	81.2	< 0.001	87.1	< 0.001	0.683
Race missing	48.6	44.9	107.5	< 0.001	188.4	< 0.001	0.001
Non-Hispanic	48.3	41.4	96.6	< 0.001	127.9	< 0.001	< 0.001
Hispanic	28.8	27.0	89.2	< 0.001	140.0	< 0.001	0.034
Hispanic missing	49.3	46.3	76.4	< 0.001	176.5	< 0.001	< 0.001
Private insurance	52.9	46.2	108.3	< 0.001	151.9	< 0.001	< 0.001
Uninsured	43.9	36.8	91.9	< 0.001	128.9	< 0.001	0.017
Medicaid	43.1	34.9	104.3	< 0.001	148.7	< 0.001	< 0.001
Medicare	45.8	39.2	88.2	< 0.001	114.4	< 0.001	< 0.001
Dual-eligible	39.4	30.6	81.2	< 0.001	103.6	< 0.001	0.001
Other insurance	46.9	41.6	92.4	< 0.001	162.0	< 0.001	< 0.001
Travel time to nearest facility (minutes)							
25th percentile	29.7	25.5	72.5	< 0.001	119.6	< 0.001	< 0.001
75th percentile	78.7	67.6	111.6	< 0.001	139.9	< 0.001	< 0.001
Chronological facility number							
25th percentile	47.6	41.1	94.4		127.2		
75th percentile	48.1	41.5	95.6		128.9		
US News hospital score							
25th percentile	41.3	38.6	51.5	< 0.001	63.7	< 0.001	< 0.001
75th percentile	69.0	57.8	189.9	< 0.001	296.7	< 0.001	< 0.001
US News hospital score missing	41.3	38.6	51.5		63.7		
Volume of cases 2010–2014 at facility attended (in 100’s)				< 0.001		< 0.001	< 0.001
25th percentile	37.2	34.3	46.5	< 0.001	60.8	< 0.001	< 0.001
75th percentile	68.7	57.6	143.6	< 0.001	198.0	< 0.001	< 0.001
Other digestive	53.6	45.7	113.5	< 0.001	166.0	< 0.001	< 0.001
Lung/bronchus	41.9	35.3	90.0	< 0.001	132.5	< 0.001	< 0.001
Colorectal	38.9	33.4	75.6	< 0.001	92.6	< 0.001	< 0.001
Prostate	58.1	51.4	101.3	< 0.001	153.0	< 0.001	< 0.001
Female breast	38.9	34.7	74.1	< 0.001	87.4	< 0.001	< 0.001
Cervical/uterine	54.5	44.9	135.7	< 0.001	171.7	< 0.001	0.143
Oral/pharyngeal	59.2	51.8	110.1	< 0.001	148.1	< 0.001	< 0.001
Ovarian	58.6	49.1	128.9	< 0.001	180.2	< 0.001	0.001
Thyroid	57.7	48.7	122.8	< 0.001	180.3	< 0.001	< 0.001
Melanoma	57.7	51.2	107.3	< 0.001	91.1	< 0.001	0.008
Other urological	50.1	41.9	102.7	< 0.001	152.2	< 0.001	< 0.001
Local	50.3	43.9	95.9	< 0.001	127.1	< 0.001	< 0.001
Regional	48.7	41.5	101.3	< 0.001	143.1	< 0.001	< 0.001
Distant	43.2	36.3	93.0	< 0.001	131.1	< 0.001	< 0.001
Unknown stage	36.7	33.6	57.4	< 0.001	57.8	< 0.001	0.951
Non-mutually exclusive treatment categories							
Chemotherapy	46.4	40.1	96.9	< 0.001	121.1	< 0.001	< 0.001
Radiation therapy	47.6	42.2	90.2	< 0.001	119.6	< 0.001	< 0.001
Surgery	49.7	42.7	99.7	< 0.001	132.6	< 0.001	< 0.001
Immunotherapy	45.6	40.9	95.1	< 0.001	93.9	< 0.001	0.898
Hormone therapy	48.5	42.5	95.4	< 0.001	115.9	< 0.001	< 0.001

**Fig. 1:**
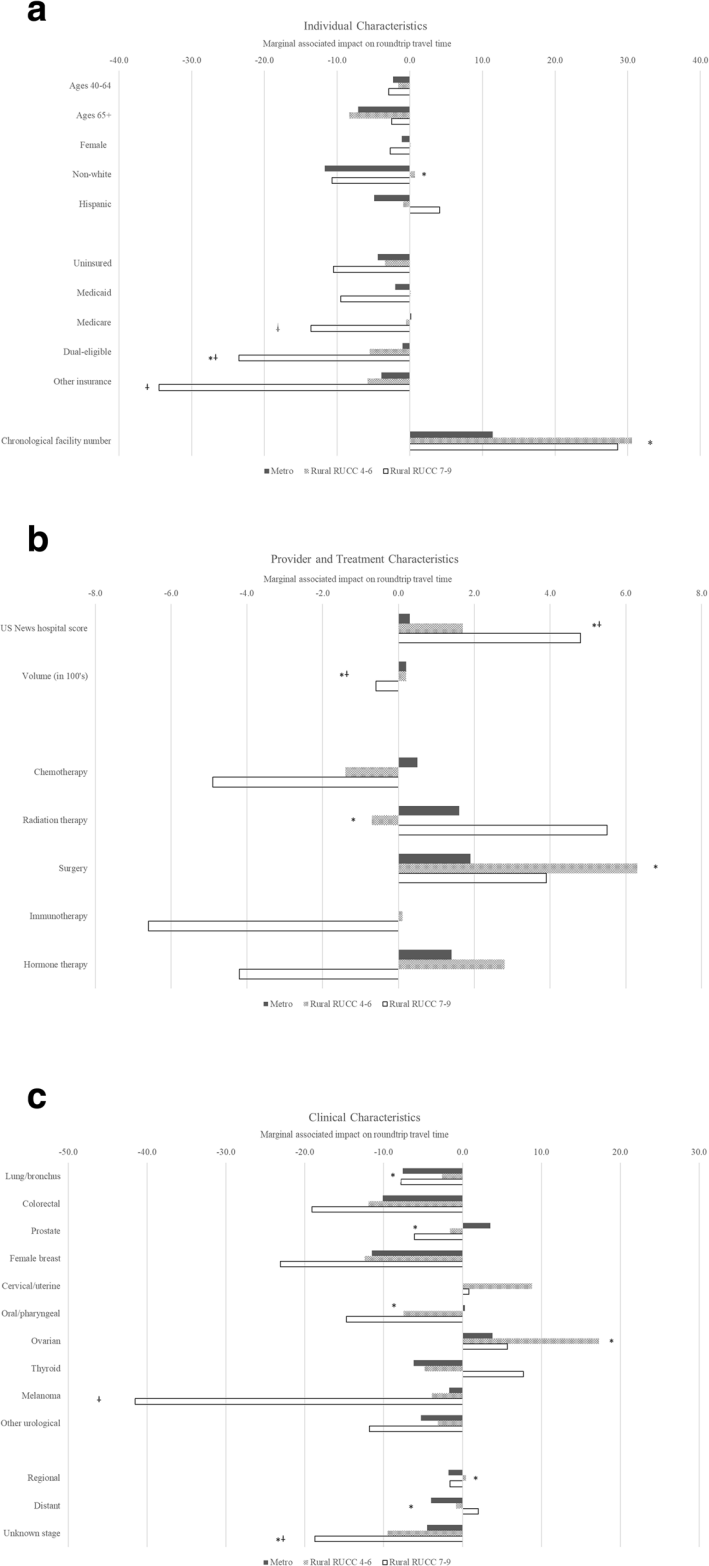
Estimated marginal contribution of individual (panel **a**), provider (panel **b**), and clinical factors (panel **c**) to roundtrip travel time fully stratified by rural-urban category. Note: The estimated value presented in the figure is the additional, marginal contribution of each factor to round trip travel time after controlling for all other listed factors based on a separate regression for each category of rurality. Standard errors are clustered at the individual level. * Represents association is statistically significantly (*p* < 0.05) for rural RUCC 4–6 patient compared to metro patient. † Represents association is statistically significantly (*p* < 0.05) for rural RUCC 7–9 patient compared to metro patient

Figure 1 shows the results from multivariate linear regressions run separately by rurality (i.e. metro, rural RUCC 4–6, and rural RUCC 7–9). In many cases, the sign of the association for each factor was similar across models with many of the magnitudes larger for the non-metro areas. However, we observed several instances where the estimated associations varied for non-metro areas relative to metro areas. For example, we found significantly more negative associations for dually Medicaid and Medicaid eligible patients—i.e. 22.5 min shorter for rural RUCC 7–9 (*p* = 0.002) and 4.5 min shorter for rural RUCC 4–6 (*p* = 0.03) relative to the association for metro patients; and for patients with unknown stage—i.e. 14.2 min shorter for rural RUCC 7–9 (*p* = 0.016) and 5.0 min shorter for rural RUCC 4–6 (*p* = 0.01) relative to the association for metro patients. Conversely, we found significantly greater travel times for both non-metro groups relative to metro patients for those attending hospitals with greater USNWR scores—4.5 min greater for each point increase in score for rural RUCC 7–9 patients (*p* < 0.001) and 1.4 min greater for each point increase for rural RUCC 4–6 patients (*p* < 0.001).

In addition, relative to metro patients we found that rural RUCC 7–9 patients with Medicare or other insurance were associated with significantly shorter travel times. For rural RUCC 4–6 patients, relative to metro patients, we found that lung/bronchus cancer, oral/pharyngeal, distant stage, and receipt of radiation therapy were all associated with relatively shorter travel times controlling for all others factors. Finally, for rural RUCC 4–6 patients, relative to metro patients, we found that being non-white, attending an additional facility, attending a higher volume hospital, having ovarian cancer, distant stage or receiving surgical treatment were all associated with significantly greater travel times controlling for all other factors.

In addition to our baseline estimates, we conducted several sensitivity analyses to examine the robustness of the results. First, we restricted the sample to hospitals that saw at least 500 patients over the 5-year period. In general, we found qualitatively very similar results (full results available upon request). In the next sensitivity analysis, we restricted the sample to just the first observed hospital for each patient to determine whether including multiple observations per patient might be affecting the results. However, this had little effect on our estimates, likely because most patients only visited a single facility (full results available upon request). Similarly, there was limited effect relative to the baseline estimates when we dropped the control variable for travel time to nearest hospital. Finally, we found evidence that NCI-designated cancer centers were associated with significantly greater travel time for all patients. In the full regression results we found that attendance at a NCI-designated cancer center was associated significantly greater travel times—controlling for all other factors we found an estimated 9.2 min longer for metro patients (*p* < 0.001), 31.1 min longer for rural RUCC 4–6 patients (*p* < 0.001), and 72.1 min longer for rural RUCC 7–9 patients (*p* < 0.001) [full results available upon request].

## Discussion

Overall, we found considerable variability in patient travel time. First, we document the magnitude of the well-known greater travel time for more rural patients [1, 22], showing mean roundtrip travel times increase from 41.5 min for metro patients to 95.6 min for rural RUCC 4–6 patients to 128.9 min for rural RUCC 7–9 patients. Relatedly, the relatively shorter times for non-white and Hispanic patients may reflect the relatively fewer racial and ethnic minorities that live in rural areas in Pennsylvania [28]. Similar to previous studies, [20, 39] we found elderly patients had relatively shorter travel times, as did those without private health insurance. Not surprisingly, patients traveled farther for hospitals with higher USNWR rankings, with greater patient volume, and to NCI-designated cancer centers, likely seeking hospitals that may be considered to be higher quality. In addition, patients often traveled farther to their second, third, or fourth hospital, consistent with patients perhaps beginning with a closer hospital but traveling farther if referred or choosing to seek care at a more distant facility.

Clinically we found important differences in travel time by cancer site, which is something providers and hospital administrators should be aware of in order to understand patient travel burden and potential need for travel assistance. For example, we consistently found that breast cancer and colorectal cancer patients had shorter travel times across each rural-urban category; and we saw mixed evidence this may also true for lung/bronchus cancer and prostate cancer. Conversely, we saw consistently greater travel times for cervical/uterine cancer and ovarian cancer as well as some mixed evidence for thyroid and other digestive cancers. Part of the explanation for the difference in travel times may be that there appear to be a greater number of providers who treat breast, colorectal, prostate, and lung/bronchus cancer (see Table 3). This suggests that providers who treat the less common cancers such as cervical/uterine, ovarian, thyroid, and other digestive cancers may need to be aware their patients, in particular, may be traveling further. We also found, consistent with the literature, that patients traveled farther for surgery but not as far for ongoing treatments, such as chemotherapy or radiation therapy [10, 11, 14].

**Table 3:** Cancer-specific hospital volume and percent of hospitals above various cancer-specific volume thresholds

Cancer site	Mean per hospital volume	Percent of hospitals with at least:			
		1 case	10 cases	50 cases	100 cases
Lung/bronchus	111	47.5%	42.1%	31.6%	26.9%
Colorectal	79	52.5%	44.0%	33.2%	26.9%
Prostate	87	50.0%	42.7%	28.5%	22.8%
Female breast	110	49.1%	43.7%	32.0%	28.8%
Cervical/uterine	5	35.4%	10.1%	3.5%	0.3%
Oral/pharyngeal	19	43.0%	27.2%	9.5%	3.8%
Ovarian	12	42.7%	19.9%	7.6%	3.5%
Thyroid	34	44.0%	28.5%	14.9%	9.8%
Melanoma	36	47.2%	32.3%	15.5%	7.9%
Other digestive	72	50.9%	40.5%	26.9%	16.1%
Other urological	60	48.4%	40.5%	26.9%	18.4%

We also found that the relationship between various characteristics and roundtrip travel time varied significantly across rural-urban categories. The greatest difference was between patients who visited more than one facility. This suggests that patients living in non-metro areas may travel considerably farther if they need to see an alternate provider, which could be necessary with a second opinion or the need to find a provider with clinical expertise not available at the first or closest hospital.

### Limitations

First, we computed average travel time by car which does not account for travel by public transportation, variations in traffic by time of day, or other factors that may variably affect travel time [40, 41]. To validate travel time, we compared a random sample of travel times to those calculated using Google maps; we observed minimal differences. Second, we were limited to the patients’ residential location at the time of diagnosis; thus, we do not have information concerning possible relocations during treatment. Third, we have limited information on patient preferences and health insurance (e.g., provider network, cost-sharing), which may be important in better understanding patients’ choices of hospitals. Because the focus of the study was more exploratory in terms of understanding factors associated with greater travel times for patients by rurality, we also note that we did not further model issues related to spatial autocorrelation. Further, while we chose to use definitions of rurality based on definitions from the literature, [32–34] we note that alterative definitions exist and that using alternative definitions could potentially affect the results. Finally, due to data limitations, we were not able to estimate the effect of travel time on survival or other outcomes.

### Strengths

Our study used population-based data, helping to minimize the potential for bias and improving generalizability as one of the first to directly explore factors that may contribute to greater travel times for rural cancer patients, a population of increasing interest to providers and policymakers. Second, our data were from Pennsylvania, the 5th most populous state, which has a heterogeneous mix of regions that provide an opportunity to study both metro and rural patients. Finally, we examined travel time for all cancer sites, thus are able to quantify important differences in travel times by cancer site and other factors.

## Conclusion

Compared to cancer patients living in more metro counties, cancer patients from non-metro counties have substantially longer travel times, which may contribute to reduced access to cancer treatment and poorer outcomes. Importantly, this difference in travel time varies across patients and cancer sites. In particular, we found patients from non-metro areas with cervical/uterine or ovarian cancer may have especially long travel times, potentially due to fewer available providers. In addition, higher volume, higher-ranked cancer hospitals should be aware that while many of their patients may live nearby, a substantial subset may be coming from non-metro areas to seek more advanced treatment and as a result may have different needs in terms of travel assistance. While our study focused on travel times once a patient has been diagnosed, the prior literature highlights that travel time and rurality may also contribute to later stage of diagnosis, further exacerbating adverse clinical outcomes in rural patients. Our results help identify these patients who may especially need assistance with travel. Importantly, hospitals and health systems should consider these differences when considering organization and patient services, including provider referral networks, patient transportation assistance, navigation programs, treatment delivery, and survivorship programs. Finally, future research is needed to continue to understand the complex interaction between rurality and travel time and its effect on receipt of timely treatment as well as cancer health outcomes.

## Data Availability

The data are not available for public release due to the data use agreement with the Pennsylvania Department of Health.

## Abbreviations

HIPAA: Health Insurance Portability and Accountability Act
NCI: National Cancer Institute
RUCC: Rural-Urban Continuum Code
USNWR: U.S. News and World Report
